# Identification of UDP-glycosyltransferases involved in the biosynthesis of astringent taste compounds in tea (*Camellia sinensis*)

**DOI:** 10.1093/jxb/erw053

**Published:** 2016-03-02

**Authors:** Lilan Cui, Shengbo Yao, Xinlong Dai, Qinggang Yin, Yajun Liu, Xiaolan Jiang, Yahui Wu, Yumei Qian, Yongzhen Pang, Liping Gao, Tao Xia

**Affiliations:** ^1^State Key Laboratory of Tea Plant Biology and Utilization, Anhui Agricultural University, Hefei, Anhui 230036, China; ^2^Key Laboratory of Plant Resources and Beijing Botanical Garden, Institute of Botany, Chinese Academy of Sciences, Beijing 100093, China; ^3^School of Life Science, Anhui Agricultural University, Hefei, Anhui 230036, China

**Keywords:** Astringent taste compounds, biosynthesis, *Camellia*, *sinensis*, flavonol 3-*O*-glycosides, galloylated catechins, UDP-glycosyltransferase.

## Abstract

Galloylated catechins and flavonol 3-*O*-glycosides are characteristic astringent taste compounds in tea (*Camellia sinensis*). The mechanism involved in the formation of these metabolites remains unknown in tea plants. In this paper, 178 *UGT* genes (*CsUGT*s) were identified in *C. sinensis* based on an analysis of tea transcriptome data. Phylogenetic analysis revealed that 132 of these genes were clustered into 15 previously established phylogenetic groups (A to M, O and P) and a newly identified group R. Three of the 11 recombinant UGT proteins tested were found to be involved in the *in vitro* biosynthesis of β-glucogallin and glycosylated flavonols. CsUGT84A22 exhibited catalytic activity toward phenolic acids, in particular gallic acid, to produce β-glucogallin, which is the immediate precursor of galloylated catechin biosynthesis in tea plants. CsUGT78A14 and CsUGT78A15 were found to be responsible for the biosynthesis of flavonol 3-*O*-glucosides and flavonol 3-*O*-galactosides, respectively. Site-directed mutagenesis of the Q373H substitution for CsUGT78A14 indicated that the Q (Gln) residue played a catalytically crucial role for flavonoid 3-*O*-glucosyltransferase activity. The expression profiles of the *CsUGT84A22*, *CsUGT78A14*, and *CsUGT78A15* genes were correlated with the accumulation patterns of β-glucogallin and the glycosylated flavonols which indicated that these three *CsUGT* genes were involved in the biosynthesis of astringent compounds in *C. sinensis*.

## Highlight

The identification of three UDP-glycosyltransferases involved in the biosynthesis of galloylated catechins and glycosylated flavonols which are astringent taste compounds in tea.

## Introduction

Polyphenols are major secondary metabolites present in tea (*Camellia sinensis*). These compounds are closely related to the typical flavour of tea infusions and to the pharmaceutical benefits of tea on human health (Thanaraj and Seshadri, 1990; Mukhtar and Ahmad, 2000; Noble, 2002; Schmidt *et al.*, 2014; Zhu *et al.*, 2014). Among these polyphenols, catechins (flavan-3-ols), flavonols, and their derivatives (galloylated catechins and flavonol 3-*O*-glycosides) are major components with pivotal bioactivities in tea. These compounds have been shown to be the major contributors to the astringent sensation of black tea infusions (Scharbert and Hofmann, 2005). In particular, galloylated catechins have been found to confer astringent and bitter tastes (Ding *et al.*, 1992; Scharbert and Hofmann, 2005; Rossetti *et al.*, 2009), while flavonol 3-*O*-glycosides have been found to induce velvety, mouth-drying, and mouth-coating sensations (Scharbert and Hofmann, 2005).

Glycosylation is an important process for the diverse functions of polyphenolic compounds in plants and is known to be able to increase the solubility and stability of hydrophobic flavonoids (Yoshida *et al.*, 2000). The glucosylation and subsequent acylation of the 3′-OH group of anthocyanins contribute to a deeper blue colour and to the stabilization of anthocyanins via intramolecular stacking (Fukuchi-Mizutani *et al.*, 2003). In addition, glycosylated polyphenols are substrates for ABC (ATP binding cassettes) or MATE (Multidrug and Toxic compound Extrusion) transporter proteins. For example, the epicatechin 3′-*O*-glucoside, formed through the glucosylation activity of UDP-glucosyltransferase UGT72L1, has been shown to be a substrate for the vacuolar transporter MATE1 and is known to be involved in proanthocyanidin biosynthesis in the seed coat of *Medicago* (Pang *et al.*, 2008; Zhao and Dixon, 2009). In addition, glycosylated polyphenols function as efficient acyl donors in biochemical reactions. For example, β-glucogallin, a glucose ester of gallic acid, functions as an efficient acyl transfer donor in the biosynthesis of both gallotannin (Niemetz and Gross, 2005) and galloylated catechins (Liu *et al.*, 2012).

**Fig. 1.**
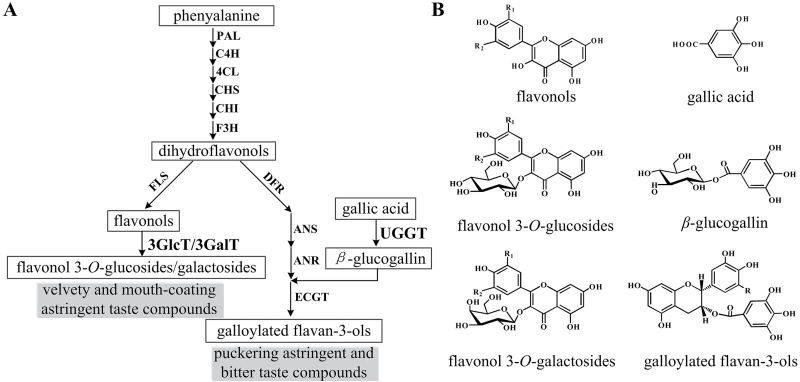
A simplified scheme showing the biosynthetic pathway from phenylalanine to astringent taste compounds in leaves of tea plants. 3GlcT, flavonol 3-*O*-glucosyltransferase; 3GalT, flavonol 3-*O*-galactosyltransferase; R=H, epicatechin gallate (ECG); R=OH, epigallocatechin gallate (EGCG); R1=H, R2=H, kaempferol/kaempferol 3-*O*-glucoside/kaempferol 3-*O*-galactoside; R_1_=H, R_2_=OH, quercetin/quercetin 3-*O*-glucoside/quercetin 3-*O*-galactoside; R_1_=OH, R_2_=OH, myricetin/myricetin 3-*O*-glucoside/myricetin 3-*O*-galactoside.

In a previous study, we found that two enzymes, UDP-glucose:galloyl-1-*O*-β-d-glucosyltransferase (UGGT) and epicatechin:1-*O*-galloyl-β-d-glucose *O*-galloyltransferase (ECGT), were involved in the two-step biosynthetic pathway of galloylated catechins in tea plants (Liu *et al.*, 2012; Fig. 1). Recent studies also confirmed that three *VvUGTs* in grapevine and one *UGT84A13* from pedunculate oak exhibited catalytic activity for the formation of 1-*O*-galloyl-β-d-glucose (β-glucogallin) (Khater *et al.*, 2012; Mittasch *et al.*, 2014). However, in tea plants, the gene(s) encoding UGGT remain as yet unknown.

In another study, we found that tea plants accumulate at least 12 flavonol glycosides with various sugar moieties at the 3-OH position, including glucose, galactose, and rhamnose (Jiang *et al.*, 2013). Although a number of UGTs have been found to catalyse reactions involving flavonols in several plant species (Ford *et al.*, 1998; Mato *et al.*, 1998; Jones *et al.*, 2003; Nagashima *et al.*, 2004; Ono *et al.*, 2010), the genes encoding flavonol 3-*O*-glycosyltransferases in tea remain uncharacterized.

The UDP-glycosyltransferases are encoded by a multigene family, which makes it difficult to screen target *UGT* genes that are specifically involved in the metabolism of astringent metabolites in tea plants. To date, more than 1 500 putative *UGTs* have been identified from various plant genomes based on the highly conserved PSPG motif. These UGT sequences have been classified into 16 distinct groups (from A to P) based on phylogenetic analysis (Caputi *et al.*, 2012). In *A. thaliana*, the UGTs responsible for the glycosylation of flavonoids, benzoates, and terpenoids are mostly found in the groups A, B, D, E, F, G, H, and L (Osmani *et al.*, 2009) and all known UGTs forming glucose esters with benzoates are of group L (Lim *et al.*, 2002). It is therefore reasonable to speculate that *UGT* genes encoding UDP-glucose:galloyl-1-*O*-β-d-glucosyltransferase (UGGT) probably belong to group L in tea.

Genome and/or transcriptome analyses have proved to be highly efficient for the discovery of candidate *UGT* genes involved in particular metabolic pathways (Gachon *et al.*, 2005; Caputi *et al.*, 2012). Recently, sequencing data from several *Camellia sinensis* transcriptome projects have become available in public databases which makes it possible to search and screen the *UGT* genes involved in the biosynthesis of β-glucogallin and flavonol 3-*O*-glycosides in tea. In this report, we describe the *in silico* identification and classification of the *CsUGT* gene family from eight transcriptome datasets. We also isolated and functionally characterized three *UGTs* that are involved in the biosynthesis of β-glucogallin and glycosylated flavonols in tea plants. We also report on the expression patterns of these three *CsUGT* genes and the content of metabolites known to confer astringent taste sensations. These analyses on the *CsUGT* gene family lay a foundation for gaining a better understanding of the CsUGT superfamily in tea and other plants.

## Materials and methods

### Chemicals

Chemical standards quercetin 3-*O*-glucoside, quercetin 3-*O*-galac toside, kaempferol 3-*O*-glucoside, kaempferol 3-*O*-galactoside, myricetin 3-*O*-galactoside, rutin, quercetin, kaempferol, myricetin, UDP-glucose, UDP-galactose, naringenin, eriodictyol, apigenin, epicatechin, cyanidin chloride, gallic acid, *p*-hydroxybenzoic acid, benzoic acid, syringic acid, cinnamic acid, *p*-coumaric acid, caffeic acid, ferulic acid, sinapic acid, uridine 5′-diphosphate disodium salt hydrate (UDP), and β-glucogallin were obtained from Sigma-Aldrich (St Louis, MO, USA). Dihydroquercetin was purchased from Shanghai Winherb Medical Technology Co., Ltd (Shanghai, China). HPLC-grade methanol, acetonitrile, and acetic acid were obtained from Tedia Co., Ltd (Fairfield, OH, USA).

### Plant materials

Plant samples (*Camellia sinensis* var. *sinensis* cv. ‘Nongkangzao’, ‘Huangjinya‘, and ‘Quntizhong’) were all collected from the horticultural research station of Anhui Agricultural University during the early spring and immediately frozen in liquid nitrogen; and samples were stored at –80 °C until use.

### Transcriptome sequencing

Transcriptome sequencing of *Camellia sinensis* var. *sinensis* cv. ‘Nongkangzao’, ‘Huangjinya‘, and ‘Quntizhong’ were performed by the BGI Gene Tech Co., Ltd. (Shenzhen, China) using the Illumina Hiseq 2000 platform. Metadata for sample information, experimental design, and unigene sequences were deposited in the Sequence Read Archive (SRA) database at NCBI under Bioproject ID PRJNA283010 for Nongkangzao, PRJNA283013 for Huangjinya, and PRJNA283232 for Quntizhong. In addition, three additional transcriptome data sets were retrieved from NCBI and they are PRJNA167330 for cultivar ‘Fudingdabaicha’, PRJNA223181 and PRJNA51793 for cultivar ‘Longjing 43’, PRJNA170701 for cultivar ‘Hongye2’, and PRJNA261465 for cultivar ‘Teenali’.

### Analysis of CsUGT gene sequences

In this study, based on the conserved PSPG motif in plant UGTs (Caputi *et al.*, 2012), most of the tea CsUGT sequences were identified using the conserved motifs ‘W-2x-Q-11x-F-2x-H-1x-GW-1x-S-9x-P-9x-Q’ and ‘W-2x-Q-3x-L-10x-H-1x-G-5x-E-17x’. Some tea CsUGT genes were identified by using sequence homology searching and by keyword (glycosyltransferase) searching.

Sequences were aligned using the ClustalW algorithm-based AlignX mode in MEGA5 (MegaSoftware, USA) (Tamura *et al.*, 2011) and a phylogenetic tree was constructed by using Neighbor–Joining distance analysis (Saitou and Nei, 1987). Tree nodes were evaluated by the Bootstrap method for 1 000 replicates (Felsenstein, 1985) and the evolutionary distances were computed using the p-distance method (Nei and Kumar, 2000).

Protein sequences of CsUGTs were applied to secondary structure prediction using the online server Jpred (http://www.compbio.dundee.ac.uk/www-jpred/). The homology models of CsUGTs were constructed using the PyMOL Molecular Graphics System with the known crystal structure as a template. Multiple sequence alignment was performed using ClustalX.

### Expression and purification of recombinant CsUGTs

Total RNA from leaves of Nongkangzao was isolated using RNAiso-mate for Plant Tissue (Takara, Dalian, China) and RNAiso Plus (Takara, Dalian, China) according to the manufacturer’s instructions. The 3′-cDNA, 5′-cDNA, and cDNA fragments were synthesized with a SMARTer™ RACE cDNA Amplification Kit (Clontech, USA) according to the manufacturer’s instructions. Full-length cDNA sequences of the *CsUGT* genes were obtained using a RACE-PCR protocol according to the manufacturer’s instructions (Clontech, USA). PCR products were purified with a MiniBEST Agarose Gel Extraction Kit (Takara, Dalian, China) and ligated into the pMD19-T simple vector and subsequently transformed into TransT1-competent cells.

The open reading frames of the *CsUGTs* were subcloned into the expression vector pMAL-c2X (New England Biolabs, MA, USA). The sequences of the cloned genes were also confirmed by sequencing with primers: pMAL-c2X-F: 5′-TGCGTACTGCGGTGATCAAC-3′ and pMAL-c2X-R: 5′-CTGCAAGGCGATTAAGTTGG-3′. All the primers used in the present study were designed with Primer Premier 5.0 software (Premier, BC, Canada) and synthesized by the Invitrogen Company (Shanghai, China). The primer sequences are listed in Supplementary Table S1 at *JXB* online.

The pMAL-c2X expression plasmids harbouring the *CsUGT* genes were transformed into *Escherichia coli* Novablue (DE3) competent cells (Novagen, Schwalbach, Germany). Recombinant proteins were purified according to the manufacturer’s instruction (New England Biolabs, MA, USA).

### Enzymatic assays of CsUGTs

To analyse the *in vitro* activity of the candidate 1-*O*-ester-forming glucosyltransferases, reactions were carried out in a 50 μl reaction solution consisting of 100mM MES buffer (pH 5.5), 2.5mM UDP-glucose (UDP-Glc) as the sugar donor, 0.5mM of phenolic acid substrates (gallic acid, *p*-hydroxybenzoic acid, benzoic acid, syringic acid, cinnamic acid, *p*-coumaric acid, caffeic acid, ferulic acid, and sinapic acid), and 6 μg of purified recombinant CsUGT protein.

To analyse the activity of flavonoid 3-*O*-glycosyltransferases, assays were performed in a buffer containing 100mM TRIS–HCl (pH 7.5), 5mM UDP-Glc or UDP-galactose (UDP-Gal) as the sugar donor, 200 μM of potential flavonoid acceptors (including quercetin, kaempferol, myricetin, naringenin, eriodictyol, apigenin, epicatechin, and cyanidin chloride), and 5–10 μg of purified recombinant CsUGT proteins.

All of these reactions were supplemented with 0.1% (v/v) β-mercaptoethanol and were performed in triplicate for 30min at 30 °C. Reaction samples lacking recombinant proteins were used as blank controls. Reactions were stopped by mixing the reaction solutions with 100% methanol (except for the reactions with cyanidin chloride which were stopped by the addition of an equal volume of 5% HCl), centrifuged, and then stored at –20 °C prior to HPLC or capillary electrophoresis (CE) analysis. The buffers for the pH test were 100mM MES from pH 4.5 to pH 6.5 and 100mM TRIS–HCl from pH 6.5 to pH 7.5. The kinetic parameters of the recombinant enzymes were obtained from hyperbolic Michalis–Menten saturation curves for substrates under optimal conditions. For the measurement of the *K*_m_ and *V*_max_ of CsUGT84A22, gallic acid and *p*-coumaric acid were used as acceptor substrates. The linear phase of the reaction was carried out in MES buffer (pH 5.5), with 2.5mM UDP-Glc, gallic acid (20 μM to 2mM) or *p*-coumaric acid (20 μM to 2mM) at 30 °C for 10min. The reaction products catalysed by CsUGT84A22 were also quantified using CE.

The *K*_m_ and *V*_max_ of CsUGT78A14 and CsUGT78A15 were determined using 5mM UDP-Glc or UDP-Gal as the sugar donor and 1.5–200 μM of flavonols as acceptors (kaempferol, quercetin, and myricetin) in TRIS–HCl buffer (pH 7.5). To analyse the kinetic parameters of sugar donors for both CsUGT78A14 and CsUGT78A15, 200 μM quercetin was used as the acceptor and 2–200 μM of UDP-Glc or UDP-Gal was used as the sugar donor. Flavonol 3-*O*-glycoside standards were used to quantify the enzymatic products. All the kinetic assays were incubated at 30 °C for 10min and repeated in triplicate.

### Qualitative and quantitative measurement of enzyme products

Products were analysed using HPLC with a Phenomenex Synergi 4 μm Fusion-RP80 column. The mobile phase consisted of 1% acetic acid in water (eluent A) and 100% acetonitrile (eluent B), with a flow rate of 1.0 ml·min^–1^. For benzoic acid derivatives (gallic acid, *p*-hydroxybenzoic acid, benzoic acid, and syringic acid), the elution programme was as follows: starting with 1% eluent B, linear gradients of 1–10% B for 0–10min, 10–12% B for 10–17min, and 12–1% B for 17–19min. For all other reactions, the elution programme of the analytical method was as follows: starting with 10% B, linear gradients from 10–15 % B for 0–5min, 15–40% B for 5–15min, 40–60% B for 15–20min, 60–80% B for 20–25min, and 80–10% B for 25–30min, followed by washing and reconditioning of the column.

Products were identified using UPLC-MS/MS as described by Jiang *et al.* (2013). Mass spectra were applied using electrospray ionization in negative ionization mode with a *m/z* range of 100~1 000. A nitrogen drying gas flow of 6.0 l·min^–1^, a desolvation temperature at 350 °C, a nebulizer pressure of 45 psi, and a capillary voltage of 3 500V were used.

To quantify the products of CsUGT84A22, UDP was measured in a CE system using a P/ACE MDQ (Beckman-Coulter, CA, USA) equipped with a diode array detector. The internal diameter (ID) of the capillary tubing was 75 cm×50 μm and the outer diameter (OD) was 375 μm. Sample injection was maintained for 8s in the CE instrument using a laboratory-made programmable arm controlled by a microcomputer via an electronic interface. A 50mM solution of H_3_BO_3_ at a pH of 8.5 (adjusted with sodium borate) was used as the elution solvent. The parameters used were as follows: applied voltage, 28kV; average current, 40 A; temperature, 30 °C; and the sample was detected at 262nm. The capillary surface was regenerated once a day by consecutively washing with 0.1M sodium hydroxide for 20min and then washing with water for 20min. Calibration graphs were obtained by injecting standard Uridine 5′-diphosphate (UDP) in the range of 20–500 μM. In all quantifications, the enzyme reactions were stopped by the addition of 100% methanol. The eluting peaks were processed using 32 Karat^TM^ Software, version 5.0 (Beckman-Coulter, CA, USA) and quantification was performed by evaluating the normalized area of UDP formed compared with the standard graph.

### Site-directed mutagenesis

Site-directed mutagenesis was performed with a gene site-directed mutagenesis kit (Biomed, Beijing, China). The plasmid pMAL-c2X harbouring CsUGT78A14 and CsUGT78A15 were used as templates to obtain the site-directed mutants of CsUGT78A14-Q373H and CsUGT78A15-H375Q, respectively. Oligonucleotide sequences specifically designed for mutagenesis are listed in Supplementary Table S1. The quantitative measurement of the recombinant enzyme products were performed using the aforementioned HPLC method.

### Quantitative analyses of astringent metabolites and validation of gene expression

Total phenolic compounds in tea plants were extracted as follows: the samples (0.2g of fresh leaves, young stems, and roots) were ground in liquid nitrogen and then sonicated in 2ml extraction solution (80% methanol with 1% hydrochloric acid) for 10min at room temperature. After centrifugation at 6 000rpm for 15min, the residues were then re-extracted twice as above. Finally, the pooled supernatant was extracted three times with an equal volume of chloroform and then centrifuged at 12 000rpm for 10min. The supernatants were all stored in –20 °C before analysis.

The qualitative and quantitative analyses of phenolic compounds were performed with a previously described UPLC-MS/MS method (Jiang *et al.*, 2013). Among these compounds, β-glucogallin, galloylated catechins (EGCG and ECG), and rutin were quantified with the corresponding standards and flavonol 3-*O*-glycosides were quantified with their relative abundance by the measurement of peak area.

The expression profiles of the *CsUGT84A22*, *CsUGT78A14*, and *CsUGT78A15* genes were characterized by qRT-PCR. The corresponding primer sequences are provided in Supplementary Table S1. The protocol for qRT-PCR and data processing were as previously described by Jiang (2013).

## Results

### Identification of CsUGT superfamily members based on transcriptome analysis

Although the genome sequencing of tea plants has not been completed yet, eight transcriptome sequencing data sets have been made available in the NCBI database. Plant tissues used for transcriptome sequencing were from tender shoots, young leaves, mature leaves, stems, young roots, flowers, flower buds, and immature seeds of two main *C. sinensis* variants, var. *sinensis* and var. *assamica*. The available transcriptome sequencing data make it possible to analyse the CsUGT superfamily in *C. sinensis*.

In this study, 178 *CsUGT* genes were identified by searching against the transcriptome data sets mentioned above. After removal of 46 partial CsUGTs (fewer than 250 amino acids in length), the remaining 132 *CsUGTs* were selected for further sequence and phylogenetic analysis (Supplementary Table S2).

Sequence analysis showed that the highly conserved amino acids were in positions 1 (W), 4 (Q), 8 (L), 10 (H), 19–24 (HCGWNS), 27 (E), 39 (P), 43 (E/D), and 44 (Q) of the PSPG consensus sequence (Supplementary Fig. S1). This is consistent with the glycosyltransferases that are known to function in the biosynthesis of plant secondary metabolites (Caputi *et al.*, 2012).

### Phylogenetic analysis of CsUGTs in tea plants

**Fig. 2.**
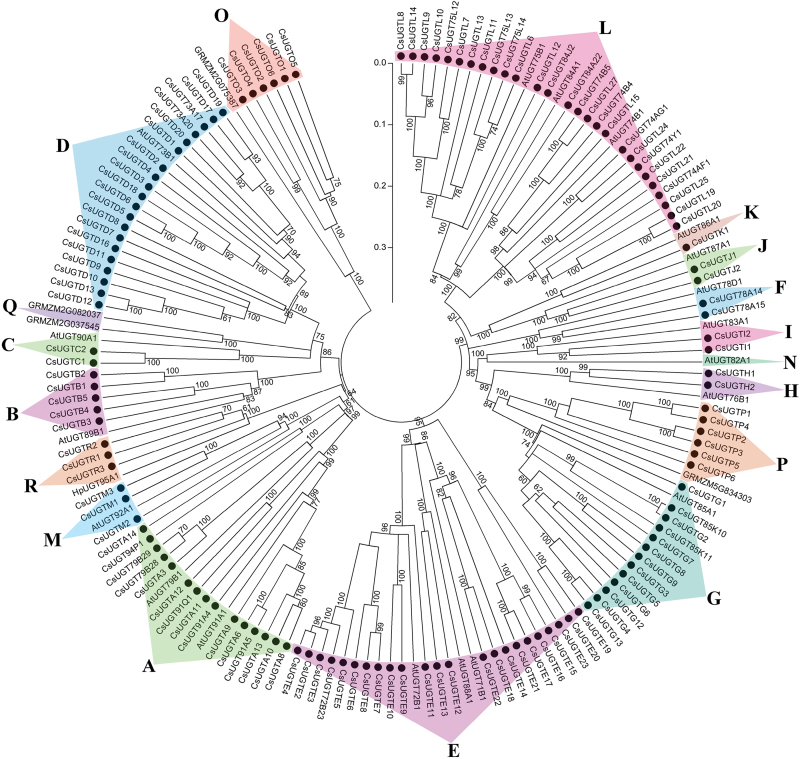
Phylogenetic tree showing clustering of 132 CsUGT family members from *C. sinensis*. The phylogenetic tree was constructed in MEGA5.0 using Neighbor–Joining and parsimony analytical methods. It contained 18 clustered groups, including groups of A–N, O–Q, and R. Bootstrap values more than 60% are indicated above the nodes. Each group is indicated with triangles and highlighted in a different colour. Sequence information of the CsUGTs is shown in Supplementary Table S2.

In a recent study, UGTs from maize (*Zea mays*) were classified into 17 groups (A–Q) (Li *et al.*, 2014). In the present study, phylogenetic analysis revealed that 132 CsUGTs could be clustered into 15 of the previously characterized groups; there were no tea UGTs in groups N and Q. Most of the CsUGTs were clustered into groups A (15), D (20), E (23), G (13), and L (27) (Fig. 2; Supplementary Table S2). Multiple sequence alignment are shown in Supplementary Table S3.

In addition, a new group, group R, was identified in our analysis and it was supported by 100% bootstrap confidence intervals (Fig. 2; Supplementary Fig. S2A). Another rooted phylogenetic tree was constructed based on amino acid sequence alignment of the CsUGTs in group R and their homologues from *V. vinifera, P. trichocarpa*, and *M. truncatula* and UGT92A1 from *A. thaliana* in group M (Fig. 2; Supplementary Fig. S2A). Results obtained indicated that group R was high divergent from group M. The MEME motif search tool showed that group R UGTs contained PSPG motifs with very highly conserved features with more than 80% identity (Supplementary Fig. S2B).

Several previously characterized UGTs in group L are known to catalyse the glucosylation of benzoates (Lim *et al.*, 2002). Nine out of 27 CsUGTs (CsUGT75L12, CsUGT75L13, CsUGT75L14, CsUGT84J2, CsUGT84A22, CsUGT74AF1, CsUGT74B4, CsUGT74B5, and CsUGT74AG1) that were scattered in different subclades of group L were selected for further analysis (Fig. 2; Supplementary Table S2). To predict the glycosylation position of these nine CsUGTs, we constructed an additional phylogenetic tree that contained several verified plant glucosyltransferases. This phylogenetic analysis showed that CsUGT75L12, CsUGT75L13, and CsUGT75L14 were clustered with flavonoid 5-*O*-glycosyltransferases, while CsUGT84J2, CsUGT84A22, CsUGT74AF1, CsUGT74AG1, CsUGT74B4, and CsUGT74B5 were grouped with other 1-*O*-ester-forming glucosyltransferases (Supplementary Fig. S3).

### Enzymatic assays and product identification of CsUGTs in group L

The open reading frames of nine CsUGTs in group L were cloned and fused to maltose-binding protein (MBP) at their N-terminus. The nine recombinant CsUGT proteins were expressed in *Escherichia coli* cells and purified for use in enzymatic assays.

To test substrate specificity, the nine purified recombinant CsUGT proteins were assayed against various phenolic compounds including flavonoids (naringenin, eriodictyol, apigenin, quercetin, kaempferol, myricetin, dihydroxykaempferol, cyanidin, and epicatechin), phenolic acid derivatives of benzoic acids (C_6_–C_1_) and cinnamic acids (C_6_–C_3_).

Among the nine recombinant CsUGT proteins, only CsUGT84A22 exhibited glucosylation activity with substrates of gallic acid, benzoic acids (*p*-hydroxybenzoic acid, benzoic acid, and syringic acid) and cinnamic acids (cinnamic acid, *p*-coumaric acid, caffeic acid, ferulic acid, and sinapic acid). The specificity of sugar donors was also evaluated using both UDP-Glc and UDP-Gal as sugar donors. The results showed that UDP-Glc was the preferred sugar donor.

**Fig. 3.**
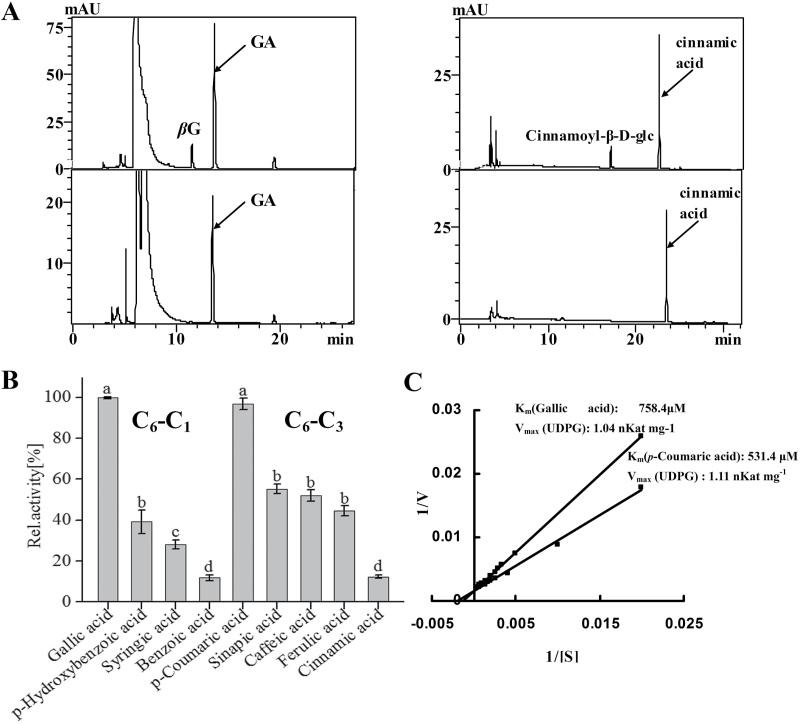
Enzymatic analysis of the recombinant UGT proteins with different substrates. (A) Representative HPLC chromatograms for enzymatic reactions with substrates gallic acid (left) and cinnamic acid (right) and their corresponding control reactions (lower panels). (B) The relative activity of CsUGT84A22 with different substrates. Assays were performed with 2mM of benzoic acids (C_6_–C_1_) or cinnamic acids (C_6_–C_3_) as acceptors and 2.5mM UDP-Glc as the sugar donor. Relative activity was referred to the reaction with gallic acid as the substrate (100%). All data presented here are the means of three replicates. Different letters above the bars indicate statistically significant differences at P <0.05, based on Tukey’s honestly significant difference test. (C) A double reciprocal plot showing 1/*V* versus 1/[gallic acid] and 1/*V* versus 1/[coumaric acid]. *K*_m_ and *V*_max_ values for recombinant CsUGT84A22 protein for these substrates are listed in the plot. All data presented are the means of three independent replicates.

HPLC analysis showed that reactions with CsUGT84A22 (Supplementary Fig. S4) generated products that were not present in the corresponding controls when gallic acid, cinnamic acid, syringic acid, *p*-coumaric acid, caffeic acid, ferulic acid, and sinapic acid were used as substrates (Fig. 3A; Supplementary Fig. S5).

UPLC-QQQ-MS/MS analysis confirmed that the enzymatic reaction product with gallic acid was 1-*O*-galloyl-β-d-glucose, identified by comparison with an authentic standard (with ms spectrum of *m/z* 331 and major ion fragments of *m/z* 169, 211, and 271) (Supplementary Fig. S5, Supplementary Table S4). The reaction products of CsUGT84A22 with other phenolic acids as substrates were identified by comparison with previously published data (Lunkenbein *et al.*, 2006; Mittasch *et al.*, 2014) (Supplementary Fig. S5, Supplementary Table S4).

To characterize the enzymatic properties of CsUGT84A22, the reactions with gallic acid as substrate and UDP-Glc as the sugar donor were conducted with pH levels ranging from 4.5–7.5 and temperatures ranging from 20–45 °C. CsUGT84A22 showed maximal activity at 30 °C and pH 5.5 and the product formed a linear gradient for at least 30min (Supplementary Fig. S6). Under these optimized reaction conditions, CsUGT84A22 had the highest catalytic activity toward gallic acid among the C_6_–C_1_ substrates and toward *p*-coumaric acid among the C_6_–C_3_ substrates (Fig. 3B). Further kinetic analysis of CsUGT84A22 revealed that it had a relatively lower *K*_m_ value for *p*-coumaric acid than for gallic acid (Fig. 3C).

### Sequence analysis of flavonol 3-*O*-glycosyltransferase genes

**Fig. 4.**
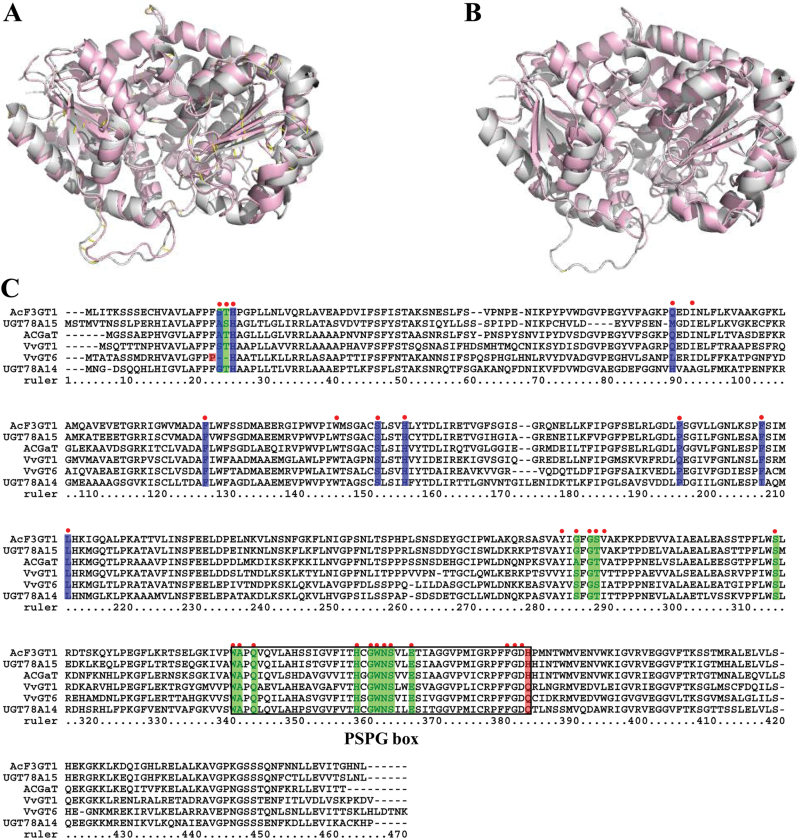
Homology modelling and multiple sequence alignment of several UGTproteins at amino acid level. (A, B) Homology models of CsUGT78A14 (A) and CsUGT78A15 (B) were constructed with 2c9z of VvGT1 as the template model. The models of VvGT1 are in grey, and CsUGT78A14 (A) and CsUGT78A15 (B) are shown in light red. (C) Multiple sequence alignment of CsUGT78A14 (KP682360) and CsUGT78A15 (KP682361) with identified UGTs including VvGT1 (AAB81683.1), VvGT6 (BAI22847.1), ACGaT (BAD06514.1), and AcF3GT1 (ADC34700.1). The multiple sequence alignment was performed using a ClustalW program. The amino acid residues for ligands are denoted with red dots, active sites for UDP-sugar donor and active sites for sugar acceptor are, respectively, highlighted in green and blue. The key residues determining the GalT and GlcT activity specificity are highlighted in red. The PSPG box of these aligned sequences is indicated by a black rectangle.

To identify specific CsUGTs encoding flavonol 3-*O*-glycosyltransferases, the secondary structure prediction of the 132 CsUGTs was carried out using the Jpred online sever (http://www.compbio.dundee.ac.uk/www-jpred). Twenty-two of these proteins had matches against the 3-D structures of UGTs that had previously been characterized, including a flavonoid 3-*O*-glucosyltransferase (2c9z_A), a triterpene/flavonoid glycosyltransferase (2acw_B), a hydroquinone glucosyltransferase (2vg8_A), or a UDP-glucuronosyl/UDP-glucosyltransferase (2pq6_A) (Supplementary Table S4). Among all of the *CsUGT*s, *CsUGT78A14* and *CsUGT78A15* displayed the highest degree of identity (56% and 53%) with VvGT1 in *V. vinifera* (crystal structure model 2c9z_A), which encodes a flavonoid 3-*O*-glycosyltransferase (Ford *et al.*, 1998) (Fig. 4A, B); this suggested that these UGTs have the highest probability of having the same function as VvGT1.

Multiple sequence alignment showed that CsUGT78A14 and CsUGT78A15 also shared relatively higher identity with several flavonoid-3-*O*-glucosyltransferases, including VvGT1, VvGT6, AcF3GT1, and AcGaT (Fig. 4C). Homology modelling analysis based on the crystal structure model 2c9z_A (VvGT1) revealed several key amino acid residues in CsUGT78A14 and CsUGT78A15 that may be involved in ligand recognition (red dots), UDP-sugar donor active sites (highlighted in green), sugar acceptor active sites (highlighted in blue), and/or the PSPG box (black rectangle) (Fig. 4C).

Within the sugar acceptor active sites, CsUGT78A14 and CsUGT78A15 differed in three residues: Gly19, His85, and Ile203 in CsUGT78A14 versus Ala23, Met82, and Phe198 in CsUGT78A15. The UDP-sugar donor active sites of these two enzymes also differed by three residues: Gly19, Ser20, and Ser280 in CsUGT78A14 and Ala23, Gly24, and Gly276 in CsUGT78A15 (Fig. 4C). It is noteworthy that the last residue of the conserved PSPG box was Q (Gln) in CsUGT78A14 and H (His) in CsUGT78A15 which are believed to be the key residues for the recognition of UDP-Glc and UDP-Gal (Gachon *et al.*, 2005), respectively, implying that CsUGT78A14 and CsUGT78A15 are able to utilize UDP-glucose and UDP-galactose as their respective sugar donors.

### Enzymatic characterization of CsUGT78A14 and CsUGT78A15

**Fig. 5.**
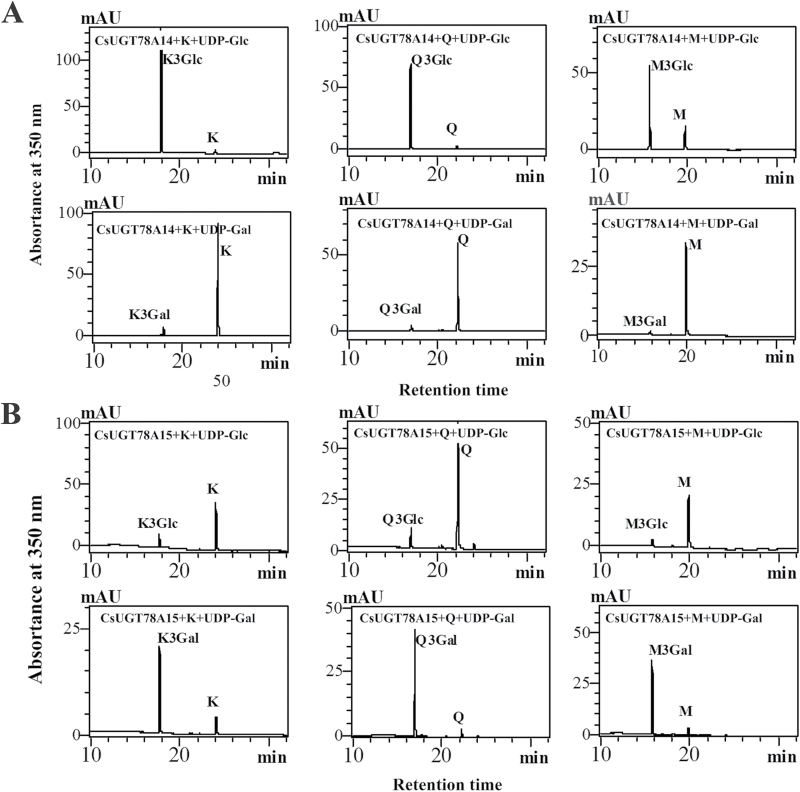
HPLC analyses of enzymatic products of the CsUGT78A14 and CsUGT78A15. (A) HPLC chromatograms for the enzymatic product of CsUGT78A14 protein with kaempferol (left), quercetin (middle), and myricetin (right) as flavonol acceptors and UDP-Glc (upper panels) or UDP-Gal (lower panels) as the sugar donor. (B) HPLC chromatograms for the enzymatic products of the CsUGT78A15 protein with kaempferol (left), quercetin (middle), and myricetin (right) as flavonol acceptors and UDP-Glc (upper panels) or UDP-Gal (lower panels) as the sugar donor.

*In vitro* enzymatic assays showed that CsUGT78A14 and CsUGT78A15 (Supplementary Fig. S4) regioselectively catalysed the glycosylation of the flavonols kaempferol, quercetin, and myricetin at the 3-OH group using both UDP-Glc and UDP-Gal as the sugar donors. HPLC-QQQ-MS/MS analysis confirmed that the enzymatic products were kaempferol-, quercetin-, and myricetin- glucosides/galactosides based on comparison with authentic kaempferol, quercetin, and myricetin 3-*O*-glycosides (Supplementary Fig. S7; Supplementary Table S4). Interestingly, CsUGT78A14 exhibited relatively stronger glucose transfer activity and relatively weaker galactose transfer activity than CsUGT78A15 (Fig. 5A), whereas CsUGT78A15 exhibited relatively lower glucose transfer activity and relatively higher galactose transfer activity (Fig. 5B). These enzymatic assay results are consistent with our prediction about sugar donor preference based on sequence analysis.

The kinetic parameters were determined for both the sugar donors and the flavonol acceptors in TRIS–HCl buffer at pH 7.5. The *K*_m_ values of UGT78A14 with myricetin, kaempferol, and quercetin were 23.7, 30.9, and 11.9 μM, with *V*_max_ values of 0.034, 0.074, and 0.036 nKat·mg^–1^, respectively (Table 1). For UGT78A15, the *K*_m_ and *V*_max_ values for myricetin, kaempferol, and quercetin were 38.8, 54.6, and 50 μM and 0.115, 0.265, and 0.184 nKat·mg^–1^, respectively. With the sugar donors, CsUGT78A14 displayed a relatively lower *K*_m_ value (14.1 μM) for UDP-Glc than CsUGT78A15 for UDP-Gal (43.3 μM) (Table 1).

**Table 1.** Kinetic parameters of recombinant CsUGT78A14 and CsUGT78A15 proteins

**Enzyme**	**Substrate**	***K***_**m**_ (μ**M**)	***V***_**max**_ **(pKat·mg** ^−**1**^)	***K***_**cat**_ **(S** ^−**1**^)	***K***_**cat**_ **/*K***_**m**_ **(S** ^−**1**^ **·M** ^−**1**^)
**CsUGT78A14**	**GlcT activity**				
	Kaempferol	30.9±3.5	73.6	6.75±0.03	0.219
	Quercetin	11.9±1.6	36.2	3.32±0.07	0.280
	Myricetin	23.8±2.1	33.9	3.11±0.04	0.131
	UDP-Glc^*^	14.1±1.8	39.3	3.61±0.02	0.257
**CsUGT78A15**	**GalT activity**				
	Kaempferol	54.6±4.5	265.1	24.49±0.83	0.448
	Quercetin	50±5.2	183.5	16.95±0.50	0.339
	Myricetin	38.8±2.3	115.2	1.06±0.12	0.027
	UDP-Gal^*^	43.3±6.5	150.5	13.90±0.95	0.321

**Fig. 6.**
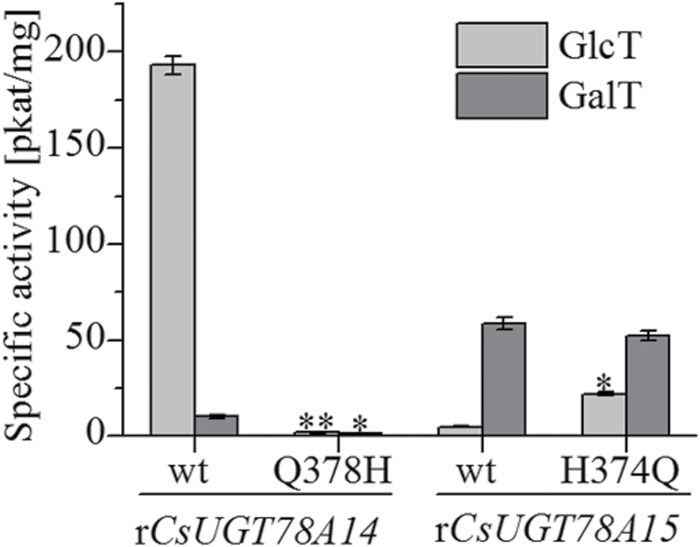
The impact of site-directed mutagenesis on the activity of CsUGT78A14 and CsUGT78A15. Data are presented as the means of three independent assays. Labelled columns are significantly different at *P* <0.05, based on Tukey’s honestly significant difference test.

To validate whether the last residue of the PSPG box is a critical determinant of the specificity of the sugar donor, site-directed mutagenesis of a Q378H substitution for CsUGT78A14 and an H374Q substitution for CsUGT78A15 were generated and the activities of the mutated proteins were analysed in enzymatic assays. The Q378H substitution of CsUGT78A14 markedly reduced the glucosyltransferase (GlcT) and galactosyltransferase (GalT) activities of CsUGT78A14, causing 99.11% and 87.75% reduction of product formation in the mutant compared with the wild-type protein. The H374Q substitution of CsUGT78A15 slightly reduced GalT activity by 10% but increased the GlcT activity by 300% (Fig. 6).

### Metabolite profiles of astringent compounds and transcript profiles of CsUGTs

**Fig. 7.**
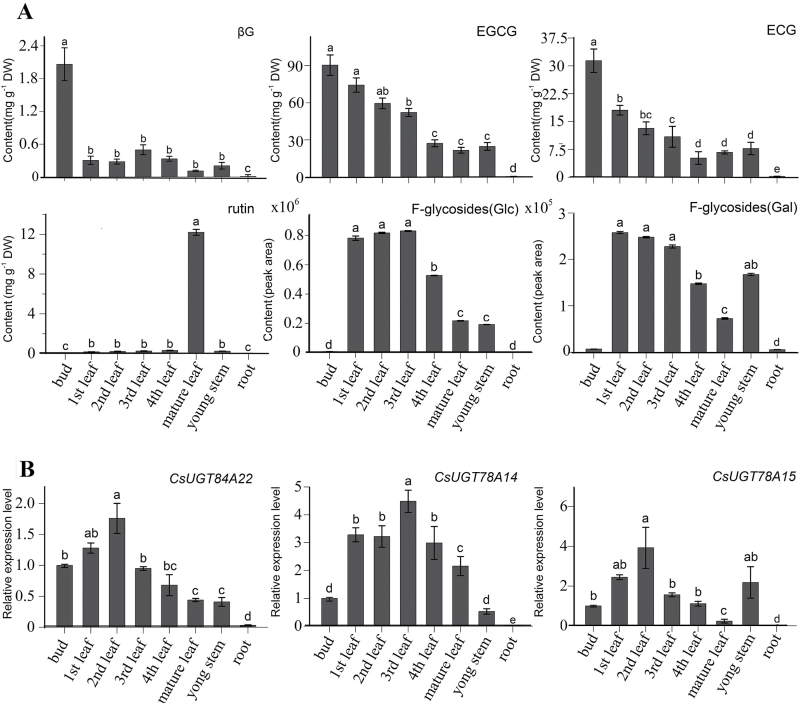
The accumulation profiles of astringent compounds and the expression patterns of three *CsUGT* genes in various organs. (A) The quantitative analysis of βG, EGCG, ECG, rutin, F-glycosides (Glc), and F-glycosides (Gal) in different organs. Levels of total F-glycosides (Glc) include kaempferol 3-*O*-glucoside, quercetin 3-*O*-glucoside, myricetin 3-*O*-glucoside, kaempferol 3-*O*-rhamnosyl-(1→6)-glucoside, kaempferol 3-*O*-[glucosyl-(1→3)-*O*-rhamnosyl-(1→6)-*O*-glucoside], quercetin 3-*O*-[glucosyl-(1→3)-*O*-rhamnosyl-(1→6)-*O*-glucoside], myricetin 3-*O*-[glucosyl-(1→3)-*O*-rhamnosyl-(1→6)-*O*-glucoside]. Levels of total F-glycosides (Gal) include kaempferol 3-*O*-galactoside, quercetin 3-*O*-galactoside, myricetin 3-*O*-galactoside, kaempferol 3-*O*-[glucosyl-(1→3)-*O*-rhamnosyl-(1→6)-*O*-galactoside], quercetin 3-*O*-[glucosyl-(1→3)-*O*-rhamnosyl-(1→6)-*O*-galactoside], and myricetin 3-*O*-[glucosyl-(1→3)-*O*-rhamnosyl-(1→6)-*O*-galactoside]. (B) The expression profiles of the *CsUGT84A22*, *CsUGT78A14*, and *CsUGT78A15* genes in different organs. All data points are the means of three biological replicates and each error bar indicates the SD. Labelled columns not connected by the same letter are significantly different at *P* <0.05, based on Tukey’s honestly significant difference test.

The major astringent taste compounds β-glucogallin, EGCG, and ECG were measured in buds, leaves, shoots, and roots by UPLC-MS/MS. The accumulation of β-glucogallin, EGCG, and ECG reached the highest levels in buds, followed by accumulation in leaves; the accumulation of these compounds was very low in roots (Fig. 7A, upper panels). The flavonol glucosides showed differential accumulation profiles. Both F-glycosides (Glc) (with the glucose directly linked to the flavonol aglycone) and F-glycosides (Gal) (with galactose directly linked to the flavonol aglycone) were more highly accumulated in buds and young leaves than in other tissues, while rutin (quercetin 3-*O*-rhamnosyl-(1→6)-β-d-glucoside) were more highly accumulated in mature leaves (Fig. 7A, lower panels).

The relative expression level of *CsUGT84A22* was measured by qRT-PCR in various tissues with the highest expression level being found in young leaves. The expression profiles of *CsUGT84A22* were not strictly consistent with the accumulation levels of β-glucogallin and galloylated catechins (Fig. 7B). The relative expression levels of *CsUGT78A14* and *CsUGT78A15* were, respectively, consistent with the accumulation patterns of F-glycosides (Glc) and F-glycosides (Gal). The accumulation of both F-glycosides (Glc) and F-glycosides (Gal) reached higher levels in young leaves than in mature leaves (Fig. 7B). Taken together, the expression profiles of the three *UGTs* genes in tea plants were largely correlated with the accumulation patterns of the major astringent taste compounds.

## Discussion

### Classification of the CsUGT superfamily in tea plants

Recently, 1 520 putative *UGT* sequences from nine plant species (*A. thaliana*, *V. vinifera*, *Glycine max*, *Sorghum bicolor*, *Oryza sativa*, *P. trichocarpa*, *Cochliobolus sativus*, *Mimulus guttatus*, and *Malus domestica*) were identified and reported. These sequences were clustered into 16 distinct groups designated groups A to P (Caputi *et al.*, 2012). In another study, 147 *UGTs* from *Z. mays* were identified and classified into 17 groups (A–Q) (Li *et al.*, 2014). Here, we found that 132 out of 178 *UGTs* of *C. sinensis* could be classified into 15 of the 17 previously described phylogenetic groups, but no *UGTs* from *C. sinensis* were found in groups N or Q.

In this study, we designated a new phylogenetic group, group R. This new group of *UGTs* comprised three members from *C. sinensis* and *UGT95A1* from *Hieracium pilosella L.* (Fig. 2; Supplementary Fig. S2); UGT95A1 had not been used for phylogenetic analysis in previous studies. All of these UGTs had highly conserved PSPG motifs (Supplementary Fig. S2). UGT95A1 had previously been shown to have highly regiospecific activity at the 3′-OH group of luteolin and quercetin and at the 7-OH group of kaempferol (Witte *et al.*, 2009). Given these results, we assume that the three CsUGTs in group R may be involved in the glycosylation of flavonoid substrates.

### Functional characterizaiton of CsUGT84A22, CsUGT78A14, and CsUGT78A15

In general, the functions of *UGT* genes can be partially predicted based on an analysis of phylogenetic relationships. For example, the *A. thaliana* UGTs responsible for the glycosylation of flavonoids, benzoates, and terpenoids are mostly clustered in groups A, B, D, E, F, G, H, and L (Osmani *et al.*, 2009). Similarly, the regioselectivity of flavonoid glycosylation can also be partially predicted based on phylogenetic relationships; the 3-*O*-, 5-*O*-, 7-*O*-glycosyltransferases and the 1,2/1,6 branch-forming glycosyltransferases form separate phylogenetic clades (Frydman *et al.*, 2013; Rodas *et al.*, 2014). Further, all of the UGTs forming glucose esters with benzoates are clustered in group L (Lim *et al.*, 2002). By using this type of approach, we hypothesized and then confirmed that the *CsUGT84A22* gene, located in group L, had specific activity toward benzoic acids and cinnamic acid derivatives (Supplementary Fig. S3).

In previous studies, some UGTs in the same clade can use a broad range of sugar acceptors *in vitro* (Hall and De Luca, 2007; Griesser *et al.*, 2008). It is also known that the UGTs that accept the same substrates are distributed in different groups of a phylogenetic tree, as is the case with UGTs from *A. thaliana* and *Z. mays* (Ross *et al.*, 2001; Li *et al.*, 2014). In this study, UGT84A22 showed specific activity toward phenolic acid derivatives of the nine UGTs assayed in group L, indicating that substrate specificity could not be solely predicted by phylogenetic analysis.

The sugar receptor and sugar donor specificity of CsUGT78A14 and CsUGT78A15 were initially predicted based on homology modelling of three-dimensional crystal structures (Supplementary Table S5) and were then verified with enzymatic analysis using recombinant proteins (Fig. 5).These experiments confirmed that this homology modelling strategy can be highly useful in the prediction of the structural features that determine substrate specificity and catalytic mechanisms (Ünligil and Rini, 2000; Hu and Walker, 2002).

### CsUGT84A22 is likely to be responsible for the biosynthesis of galloylated catechins

β-glucogallin is not only a galloyl acceptor in the biosynthesis of hydrolysable tannins but is also the activated galloyl donor during the process of galloyl transfer in the biosynthesis of both hydrolysable tannins (Niemetz and Gross, 2005) and galloylated catechins (Liu *et al.*, 2012).

In grape, three UGTs, VvgGT1, VvgGT2, and VvgGT3, catalyse the biosynthesis of β-glucogallin (Khater *et al.*, 2012). In addition, UGT84A13 in pedunculate oak was found to be the enzyme catalysing the first committed step of gallotannin biosynthesis (Mittasch *et al.*, 2014). We speculated that one or more *UGT* genes might be involved in the biosynthesis of β-glucogallin in tea plants. In this paper, the *CsUGT84A22* gene was successfully identified and functionally characterized as a *UGGT* gene that is responsible for the biosynthesis of β-glucogallin in tea plants (Fig. 3).

*In vitro* kinetic assays with the three purified recombinant VvgGT enzymes revealed that they exhibited specific activity towards both C_6_–C_1_ and C_6_–C_3_ compounds and exhibited a higher affinity for caffeic acid (C_6_–C_3_) than for gallic acid (C_6_–C_1_). By comparison, UGT84A13 from pedunculate oak exhibited a preference for C_6_–C_1_ acids and had a higher affinity for vanillic acid and protocatechuic acid than for gallic acid (C_6_–C_1_) (Mittasch *et al.*, 2014). Compared with the VvgGTs and UGT84A13, CsUGT84A22 in the present study showed the highest catalytic activity towards gallic acid among the various benzoic acid derivatives tested and showed the highest catalytic activity towards *p*-coumaric acid among the various cinnamic acid derivatives under optimized reaction conditions (Fig. 3B). CsUGT84A22 exhibited a higher affinity for *p*-coumaric acid than for gallic acid (Fig. 3C). β-Glucogallin, was detected in tea plants in a previous study (Jiang *et al.*, 2013), and was also detected in the present study (Fig. 7A). The expression profile of the *CsUGT84A22* gene did not appear to be correlated with the accumulation pattern of β-glucogallin and galloylated catechins (Fig. 7B, C) which may indicate that their accumulation is affected not only by glucosylation alone but also by some other factors, such as galloylation and/or hydrolysis (Jiang *et al.*, 2013). In addition, the glucosylated cinnamic acid derivatives have not yet been identified in tea plants and their physiological significance remains to be characterized.

### CsUGT78A14 and CsUGT78A15 are likely to be responsible for the biosynthesis of flavonol 3-*O*-monoglycoside

Flavonol 3-*O*-glycosides, as the compounds responsible for the velvety astringent flavour of tea infusions, are therefore important phenolic compounds in tea plants. Among these compounds,quercetin 3-*O*-galactoside, quercetin 3-*O*-glucoside, and kaempferol 3-*O*-glucoside showed lower threshold concentrations and higher impacts for astringent taste than did flavan-3-ols (Scharbert and Hofmann, 2005).

At least 12 flavonol 3-*O*-glycosides, including flavonol 3-*O*-monoglycosides, flavonol 3-*O*-diglycosides, and flavonol 3*-O*-triglycosides with glucose, galactose or rhamnose as the glycosyl group, have been detected in shoots of tea plants (Jiang *et al.*, 2013; Fig. 7). The expression profiles of the *CsUGT78A14* and *CsUGT78A15* genes were correlated with the accumulation patterns of F-glycosides (Glc) and F-glycosides (Gal), respectively (Fig. 7). These results implied that CsUGT78A14 and CsUGT78A15 are likely to be involved in the biosynthesis of astringent flavonol 3-*O*-monoglycoside compounds in tea plants.

Our enzymatic assays confirmed that both CsCsUGT78A14 and CsCsUGT78A15 had catalytic activity as a flavonol 3-*O*-glucosyltransferase and as a flavonol 3-*O*-galactosyltransferase (Fig. 5). The CsUGT78A14 and CsUGT78A15 proteins produced both flavonol 3-*O*-glucosides and flavonol 3-*O*-galactosides, respectively, in *in vitro* assays (Fig. 5). The site-directed mutagenesis of the Q378H substitution of CsUGT78A14 and the H374Q substitution of CsUGT78A15 showed that the Q378 residue plays an important role as a substrate-binding residue for UDP-glucose, whereas the H374 residue may not be the only key residue in CsUGT78A15 (Fig. 6), which is different from the site-directed mutagenesis of H375Q in ACGaT that altered the sugar donor specificities by this single point mutation (Kubo *et al.*, 2004).

In tea plants, flavonol 3-*O*-diglycosides such as rutin (querctin 3-*O*-rhamnosyl-(1→6)-glucoside) are able to induce a velvety and mouth-coating sensation at much lower threshold concentrations than are the flavonol 3-*O*-monoglycosides (Scharbert and Hofmann, 2005). The present results show that rutin was accumulated at low levels in shoots but at a very high level in mature leaves (Fig. 7B). However, the biosynthesis of diglycosides and the gene(s) encoding UDP-rhamnose: flavonol 3-*O*-glucoside-(1→6)-rhamnosyltransferases remain to be characterized in tea plants. To date, several *UGTs* encoding flavonoid glucoside-(1→6)-rhamnosyltransferases have been characterized in other plant species, such as the *Rt* gene for an anthocyanin 3-*O*-glucoside(1→6)-rhamnosyltransferase in *Petunia* (Kroon *et al.*, 1994), the *Cs16RhaT* gene for a flavanone 7-*O*-glucoside (1→6) rhamnosyltransferase in orange (Frydman *et al.*, 2013), and the *GmF3G6Rt* gene for a flavonol 3-*O*-glucoside (1→6) rhamnosyltransferase in soybean (Rodas *et al.*, 2014). All of these *UGTs* belong to a cluster that can catalyse the formation of 1,2/1,6 sugar branches and this information will be valuable for the identification of *UGT* genes encoding flavonol 3-*O*-glucoside-(1→6)-rhamnosyltransferase in tea plants in the near future.

## Supplementary data

Supplementary data can be found at *JXB* online.

Table S1. The primer sequences used in this study.

Table S2. Sequences information used in the phylogenetic tree in Fig. 2.

Table S3. Multiple sequence alignment of protein sequences used for the phylogenetic tree construction in Fig. 2.

Table S4. Identification of reaction products of three recombinant CsUGTs using HPLC-MS/MS analyses.

Table S5. The CsUGTs screened using a secondary structure prediction server.

Fig. S1. PSPG motif of CsUGTs.

Fig. S2. Phylogenetic analysis and PSPG motif of the group R.

Fig. S3. The position divergence of glycosylation for nine CsUGTs in group L and several UGTs from other plant species.

Fig. S4. SDS-PAGE analysis of protein extracts from *E. coli* expressing CsUGT-maltose binding protein fusion.

Fig. S 5. HPLC charts (upper), mass spectrum (middle), and MS2 spectrum (lower) of the enzymatic products catalysed by rCsUGT84A22 with gallic acid (A), syringic acid (B), cinnamic acid (C), *p*-coumaric acid (D), caffeic acid (E), ferulic acid (F), or sinapic acid (G) as substrates.

Fig. S6. Temperature and pH value optimization for the activity of rCsUGT84A22 with gallic acid and UDP-glucose as substrates.

Fig. S7. Mass and MS/MS spectra of enzymatic products of rCsUGT78A14 and rCsUGT78A15 enzymes.

Supplementary Data
