# Supplementary material for: Identification of UDP-glycosyltransferases involved in the biosynthesis of astringent taste compounds in tea (Camellia sinensis)
Source: J Exp Bot. 2016 Mar 2;67(8):2285–97. doi: 10.1093/jxb/erw053 (PMC4809296; doi:10.1093/jxb/erw053)
Supplement: Supplementary Data [file supp_67_8_2285__index.html]

Identification of UDP-glycosyltransferases involved in the biosynthesis of astringent taste compounds in tea (Camellia sinensis) — Identification of UDP-glycosyltransferases involved in the biosynthesis of astringent taste compounds in tea (Camellia sinensis) — Supplementary Data 

# Identification of UDP-glycosyltransferases involved in the biosynthesis of astringent taste compounds in tea (*Camellia sinensis*)

## Supplementary Data

Data files

- supplementary\_tables\_S1\_S2\_\_S4\_S5\_figures\_S1\_S7.pdf - Supplementary Data
- supplementary\_table\_S3.xls - Supplementary Data
