# Supplementary material for: Identification of UDP-glycosyltransferases involved in the biosynthesis of astringent taste compounds in tea (Camellia sinensis)
Source: J Exp Bot. 2016 Mar 2;67(8):2285–97. doi: 10.1093/jxb/erw053 (PMC4809296; doi:10.1093/jxb/erw053)
Supplement: Supplementary Data [file supp_erw053_supplementary_tables_S1_S2__S4_S5_figures_S1_S7.pdf]

Supplementary Data

Supplementary Table S1. The primer sequences used in this study.

purpose	primer name	sequence 5'-3'
**Protein expression**	UGT75L12-pMAL-F	GGATCCATGGTTCACAAGCACATCCTCC
	UGT75L12-pMAL-R	GTCGACTTAAACCTCATCAACGAAAGCCC
	UGT75L13-pMAL-F	TCTAGAATGCAAGCGGAGAAAGCTAGGCAGC
	UGT75L13-pMAL-R	CTGCAGCTATAAGCAATCTCCTCCAACCTCCTCCAC
	UGT75L14-pMAL-F	TCTAGAATGGGTGTCAAGGTCACCCTAGTCACC
	UGT75L14-pMAL-R	CTGCAGTTAAGACTTGTCAAAAAAAATTTCAACC
	UGT84A22-pMAL-F	TCTAGAATGGGCTCTGAATCACTTGTCC
	UGT84A22-pMAL-R	CTGCAGTTAAACAACAGTAGTAGTTGTG
	UGT84J2-pMAL-F	GGATCCATGGCTGCCATGGAAAAGAAAGAAGAG
	UGT84J2-pMAL-R	CTGCAGTCACGTACTAGAATCCCCAATTATCTCATCC
	UGT78A14-pMAL-F	TCTAGAATGAACGGTGACTCCCAACAACACC
	UGT78A14-pMAL-R	GTCGACTTAAGGGTGCTTACAAGCTTTGATTACC
	UGT78A15-pMAL-F	TCTAGAATGTCGACGATGGTGACTAACTCCTC
	UGT78A15-pMAL-R	CTGCAGTCAAAGATTGAGACTTGTTACCACC
	UGT74AG1-pMAL-F	TCTAGAATGGCGGAAGAGACAGCTTAC
	UGT74AG1-pMAL-R	CTGCAGTTAAGCAGCTGCAAGGCAAG
	UGT74AF1-pMAL-F	TCTAGAATGGAGACGAAGGAAAAGAATCC
	UGT74AF1-pMAL-R	CTGCAGCTAGGAACATAATAATTCAGC
	UGT74B4-pMAL-F	TCTAGAATGGAGCCAAAAAACCATGG
	UGT74B4-pMAL-R	CTGCAGCTATCCTTTGTTATTACTAC
	UGT74B5-pMAL-F	TCTAGAATGGAGCAAAGAAACCATGGAGG
	UGT74B5-pMAL-R	CTGCAGTCAATAATGACCATTTCCATTCC
**Site-directed mutagenesis**	UGT78A14-Q373H-F	CCGTTCTTTGGAGATCACACATTGAATAGTCGTATG
	UGT78A14-Q373H-R	CATACGACTATTCAATGTGTGATCTCCAAAGAACGG
	UGT78A15- H375Q-F	CCATTCTTTGGGGATCAACATATCAACACATGG
	UGT78A15- H375Q-R	CCATGTGTTGATATGTTGATCCCCAAAGAATGG

**Real time PCR**	UGT84A22-F	ATCAAGTGACCGATGCCAAGT
	UGT84A22-R	CGTTCGCCTTCATCTCTGCTG
	UGT78A14-F	ATGAGACTGGCTGCCTGTTG
	UGT78A14-R	CTGAGTGACCAAAGGAACGG
	UGT78A15-F	TTGGTGTGAGAGTTGAGGG
	UGT78A15-R	GGTCCAACAGCCTTGAGAG

Supplementary Table S2. Sequences information used in the phylogenetic tree (Figure 2).

Group	Unigenes	NO.	Match in NCBI	Length	Designation	Function prediction/description
A	CL7924.Contig2_All	CsUGTA1	HP759546.1	(1383bp)	CsUGT79B28	AtUGT79B1:Anthocyanidin 3-*O*-glucoside 2'''-*O*-xylosyltransferase
		CsUGTA2	GAAC01027635.1	(1383bp)	CsUGT79B29	
	CL5727.Contig1_All	CsUGTA3	GBBZ01012627.1	1374bp		
	CL3271.Contig3_All	CsUGTA4	GBBZ01003660.1	(1410bp)	CsUGT91Q1	
	Unigene14993_All	CsUGTA5	GBBZ01000629.1	(1452bp)	CsUGT91A4	
	CL5059.Contig6_All	CsUGTA6	GBBZ01042430.1	1374bp		
		CsUGTA7	HP746281.1	(1464bp)	CsUGT91A5	
	CL5059.Contig1_All	CsUGTA8	HP733423.1	1422bp		
	CL5059.Contig12_All	CsUGTA9	HP740237.1	1461bp		
	Unigene7122_All	CsUGTA10	GBBZ01007221.1	1428bp		
	Unigene22922_All	CsUGTA11	HP705742.1	1437bp		
	Unigene19016_All	CsUGTA12	GBBZ01000543.1	1416bp		
	CL5059.Contig13_All	CsUGTA13	GBHI01072771.1	1383bp		
		CsUGTA14	HP738805	1344bp		
		CsUGTA15	AB847093.1	(1362bp)	CsUGT94P1	
B	CL10999.Contig1_All	CsUGTB1	GAAC01043999.1	1365bp		AtUGT89B1: Flavonol 3-*O*-glucosyltransferase/Flavonol 7-*O*-glucosyltransferase; AtUGT89C1: Flavonol 7-*O*-rhamnosyltransferase
	CL5465.Contig4_All	CsUGTB2	HP752150.1	1497bp		
	CL3752.Contig1_All	CsUGTB3	HP745596.1	1416bp		
	Unigene9912_All	CsUGTB4	HP736425.1	1248bp		
	Unigene27742_All	CsUGTB5	HP756847.1	1425bp		
C	CL7925.Contig1_All	CsUGTC1	HP765019.1	1416bp		
	CL218.Contig4_All	CsUGTC2	N	1407bp		
D	Unigene15896_All	CsUGTD1	KA297711.1	1470bp		AtUGT73B2: Flavonol 7-O-glucosyltransferase; AtUGT73B3、
	CL9730.Contig1_All	CsUGTD2	HP746171.1	1443bp		

	CL3360.Contig4_All	CsUGTD3	HP732624.1	1473bp		AtUGT73B4、AtUGT73B5: Flavonol 3-*O*-glucosyltransferase; AtUGT73C1、AtUGT73C5: Cytokinin-*O*-glucosyltransferase;AtUGT73C6: Flavonol-3-*O*-glycoside-7-*O*-glucosyltransferase 1/Zeatin *O*-glucosyltransferase
	CL1217.Contig4_All	CsUGTD4	HP738370.1	861bp		
	CL1217.Contig2_All	CsUGTD5	GBBZ01000955.1	1506bp		
	CL1217.Contig1_All	CsUGTD6	HP724285.1	1521bp		
	Unigene31252_All	CsUGTD7	GBBZ01000579.1	1461bp		
	CL6572.Contig1_All	CsUGTD8	GBBZ01002244.1	1482bp		
	Unigene18413_All	CsUGTD9	GAAC01013915.1	1137bp		
	CL6203.Contig1_All	CsUGTD10	KA285382.1	1506bp		
		CsUGTD11	HP734430.1	1503bp		
	CL6203.Contig2_All	CsUGTD12	GBBZ01027482.1	1497bp		
	CL7509.Contig1_All	CsUGTD13	HP769577.1	1122bp		
		CsUGTD14	AB847095.1	(1422bp)	CsUGT73A17	
		CsUGTD15	GAAC01010253.1	(1428bp)	CsUGT73A20	
	Unigene21270_All	CsUGTD16	HP767350.1	1470bp		
	CL6066.Contig5_All	CsUGTD17	GAAC01037373.1	1443bp		
	CL5985.Contig2_All	CsUGTD18	GBBZ01007909.1	1362bp		
	CL6066.Contig3_All	CsUGTD19	GBBZ01009056.1	1434bp		
	Unigene22175_All	CsUGTD20	HP748188.1	1107bp		
	CL599.Contig3_All	CsUGTE1	GBBZ01010952.1	(1398bp)	CsUGT72B23	
	CL599.Contig4_All	CsUGTE2	GBBZ01011261.1	1398bp		
		CsUGTE3	GBBZ01010952.1	1398bp		
	CL5750.Contig4_All	CsUGTE4	KA291757.1	1398bp		
	CL599.Contig5_All	CsUGTE5	GBBZ01022194.1	1404bp		AtUGT72B1: Probable hydroquinone glucosyltransferase; AtUGT72E2、AtUGT72E3: Hydroxycinnamate 4-*β*-glucosyltransferase
**E**	CL8044.Contig1_All	CsUGTE6	KA286997.1	1413bp		
	CL2139.Contig1_All	CsUGTE7	GBBZ01001717.1	1422bp		
	CL2139.Contig7_All	CsUGTE8	GBRC01013013.1	1422bp		
	CL2139.Contig2_All	CsUGTE9	HP753644.1	1413bp		
	Unigene303_All	CsUGTE10	GAAC01047670.1	1104bp		
	CL5607.Contig1_All	CsUGTE11	KA290025.1	1452bp		

	CL8329.Contig1_All	CsUGTE12	HP744216.1	1440bp		
	Unigene25561_All	CsUGTE13	GBBZ01003486.1	1434bp		
	CL8176.Contig1_All	CsUGTE14	GBBZ01026787.1	1461bp		
	CL1111.Contig10_All	CsUGTE15	KA302663.1	1440bp		
	CL4578.Contig3_All	CsUGTE16	KA302663.1	1440bp		
	CL1111.Contig1_All	CsUGTE17	HP758318.1	876bp		
	CL10052.Contig2_All	CsUGTE18	HP738272.1	840bp		
	CL5284.Contig1_All	CsUGTE19	GAAC01005500.1	1443bp		
	CL5284.Contig2_All	CsUGTE20	GAAC01011341.1	1443bp		
	CL4578.Contig4_All	CsUGTE21	KA281847.1	1401bp		
		CsUGTE22	GAAC01049585.1	750bp		
	Unigene19632_All	CsUGTE23	HP739690.1	1386bp		
**F**	Unigene2202_All	CsUGTF1	GAAC01051887.1	(1380bp)	CsUGT78A14	AtUGT78D1: Flavonol-3-*O*-glucoside L-rhamnosyltransferase; AtUGT78D2: Flavonoid 3-*O*-glucosyltransferase
	CL3506.Contig2_All	CsUGTF2	GBBZ01000635.1	(1365bp)	CsUGT78A15	
	CL8533.Contig1_All	CsUGTG1	HP754065.1	1443bp		
	CL2293.Contig2_All	CsUGTG2	GBBZ01000626.1	1443bp		
	Unigene185_All	CsUGTG3	HP741294.1	1458bp		
	Unigene5928_All	CsUGTG4	GAAC01032972.1	909bp		
	CL865.Contig1_All	CsUGTG5	KA298416.1	1455bp		
	CL2663.Contig5_All	CsUGTG6	GAAC01013130.1	1416bp		AtUGT85A1: Cytokinin-*O*-glucosyltransferase /Zeatin *O*-glucosyltransferase
**G**	Unigene26015_All	CsUGTG7	HP721229.1	843bp		
	CL3081.Contig3_All	CsUGTG8	HP738451.1	1434bp		
	CL3081.Contig4_All	CsUGTG9	GBBZ01003620.1	1455bp		
		CsUGTG10	AB847092.1	(1458bp)	CsUGT85K11	
		CsUGTG11	AB847091.1	(1443bp)	CsUGT85K10	
	CL7513.Contig1_All	CsUGTG12	GBBZ01000393.1	1215bp		
	CL1241.Contig1_All	CsUGTG13	HP761406.1	1503bp		

H	Unigene8707_All	CsUGTH1	GBBZ01042418.1	1359bp		ATUGT76C1、ATUGT76C2: Cytokinin-*N*-glucosyltransferase
	CL2433.Contig1_All	CsUGTH2	GBRC01006600.1	1395bp		
I	CL8473.Contig2_All	CsUGTI1	KA295586.1	1380bp		
	CL10119.Contig2_All	CsUGTI2	HP706329.1	804bp		
J	CL6335.Contig1_All	CsUGTJ1	GBBZ01009372.1	1377bp		
	CL63.Contig1_All	CsUGTJ2	GBRC01009489.1	1389bp		
K	Unigene11927_All	CsUGTK1	KA294108.1	1512bp		
L	Unigene26360_All	CsUGTL1	KA279970.1	(1464bp)	CsUGT84A22	AtUGT84A1、AtUGT84A3、AtUGT84A4: Hydroxycinnamate glucosyltransferase; AtUGT84A2: Sinapate 1-glucosyltransferase; AtUGT75B1、AtUGT75B2: Indole-3-acetate *β*-glucosyltransferase; AtUGT75C1: Anthocyanin 5-*O*-glucosyltransferase AtUGT74B1: *N*-hydroxythioamide S-*β*-glucosyltransferase; AtUGT74D1: Jasmonate glucosyltransferase 1; AtUGT74F1: Flavonol 7-*O*-glucosyltransferase
	Unigene8605_All	CsUGTL2	HP741442.1	(1434bp)	CsUGT84J2	
	Unigene28536_All	CsUGTL3	HP739284.1	(1419bp)	CsUGT75L12	
	CL3177.Contig1_All	CsUGTL4	HP712949.1	(1419bp)	CsUGT75L13	
	CL3177.Contig2_All	CsUGTL5	HP763006.1	(1386bp)	CsUGT75L14	
	Unigene237_All	CsUGTL6	GAAC01005310.1	1014bp		
	CL4507.Contig6_All	CsUGTL7	HP730490.1	942bp		
	CL3407.Contig1_All	CsUGTL8	GAAC01049574.1	1416bp		
	CL4507.Contig4_All	CsUGTL9	GBBZ01030069.1	1386bp		
	Unigene14385_All	CsUGTL10	N	771bp		
	CL2234.Contig2_All	CsUGTL11	KA288829.1	1392bp		
	Unigene31845_All	CsUGTL12	GAAC01014189.1	1119bp		
	CL145.Contig2_All	CsUGTL13	HP767723.1	999bp		
		CsUGTL14	HP772430.1	948bp		
	CL9144.Contig1_All	CsUGTL15	HP753021.1	1380bp		
	CL9531.Contig1_All	CsUGTL16	GBBZ01016238.1	(1380bp)	CsUGT74B4	
	CL9531.Contig2_All	CsUGTL17	HP767926.1	(1416bp)	CsUGT74B5	
	CL8546.Contig3_All	CsUGTL18	GBBZ01001192.1	(1407bp)	CsUGT74AF1	
	CL10612.Contig1_All	CsUGTL19	GBRC01032560.1	1263bp		
	CL4351.Contig1_All	CsUGTL20	HP749326.1	1263bp		
	CL8116.Contig2_All	CsUGTL21	KA290501.1	1398bp		
	CL3169.Contig2_All	CsUGTL22	GAAC01050421.1	1071bp		

		CsUGTL23	KP682370	(1368bp)	CsUGT74AG1	
	CL725.Contig1_All	CsUGTL24	N	1368bp		
	CL10612.Contig2_All	CsUGTL25	HP744877.1	1383bp		
		CsUGTL26	AB847094.1	(1392bp)	CsUGT74Y1	
	CL5526.Contig4_All	CsUGTL27	GBRC01008315.1	1035bp		
**M**	CL9387.Contig2_All	CsUGTM1	HP729969.1	1467bp		
	Unigene2534_All	CsUGTM2	HP760325.1	1341bp		
	Unigene27150_All	CsUGTM3	HP732420.1	1485bp		
**O**	Unigene37295_All	CsUGTO1	N	780bp		
	CL10788.Contig1_All	CsUGTO2	GBBZ01004574.1	1389bp		
	CL10224.Contig2_All	CsUGTO3	GBBZ01004994.1	1425bp		
	CL10224.Contig1_All	CsUGTO4	HP716467.1	1332bp		
	CL8628.Contig1_All	CsUGTO5	HP731595.1	1344bp		
		CsUGTO6	GAAC01000807	1107bp		
**P**	CL2668.Contig4_All	CsUGTP1	GAAC01049718.1	1479bp		
	CL2668.Contig2_All	CsUGTP2	KA291927.1	1359bp		
	CL10732.Contig5_All	CsUGTP3	GAAC01034240.1	1443bp		
		CsUGTP4	HP758887.1	1440bp		
	CL3413.Contig3_All	CsUGTP5	KA281433.1	1380bp		
	Unigene17482_All	CsUGTP6	GBRC01011103.1	1152bp		
**R**	Unigene19430_All	CsUGTR1	HP755752.1	1443bp		
	Unigene32657_All	CsUGTR2	KA282566.1	1569bp		Flavonoid glycosyltransferase
	Unigene24589_All	CsUGTR3	GAAC01049398.1	1407bp		

Note: The CsUGTs with full-length are indicted by ().

Supplementary Table S4. Identification of reaction products of three recombinant CsUGTs using HPLC-MS/MS analyses

Products	t_{R} (min)	UV λ_{max} (nm)	MS[M-H]$^-$ (m/z)	MS$^-$/MS$^-$ (m/z)
		**rCsUGT84A22**		
Galloyl-*β*-D-glucose	10.77	277	331	169,211,271
Syringoyl-*β*-D-glucose	9.14	282	359	197,239,299
Cinnamoyl-*β*-D-glucose	17.29	282	309	109,147,236
p-Coumaroyl-*β*-D-glucose	12.88	315	325	145,163,265
Caffeoyl-*β*-D-glucose	9.7	330	341	161,179,281
Feruloyl-*β*-D-glucose	13.68	330	355	193,217,235
Sinapoyl-*β*-D-glucose	13.46	332	385	190,205,223
		**rCsUGT78A14 and rCsUGT78A15**		
Kaempferol 3-*O*-glucoside	17.98	348	447	227,255,284
Kaempferol 3-*O*-galactoside	17.72	348	447	227,255,284
Quercetin 3-*O*-glucoside	17.05	355	463	271,300
Quercetin 3-*O*- galactoside	17.03	355	463	271,300
Mycertin 3-*O*-glucoside	15.82	356	479	151,317
Myricetin 3-*O*- galactoside	15.76	356	479	151,317

Supplementary Table S5. The CsUGTs screened using a secondary structure prediction server.

No	CsUGTs	Score	Expect	Identities	Positives	Gaps	Crystal model	Function prediction
1	UGT78A14	504 bits (1297)	E-142	56%	70%	0%	2c9z_A mol	Flavonoid
2	UGT78A15	481 bits (1238)	E-135	53%	69%	2%	2c9z_A mol	3-*O*-glucosyltransferase
3	CsUGTE14	470 bits (1209)	E-132	52%	67%	8%	2acw_B mol	Multifunctional triterpene/flavonoid glycosyltransferase
4	UGT72B23	531 bits (1367)	E-150	57%	75%	1%	2vg8_A mol	
5	CsUGTE2	523 bits (1348)	E-148	56%	75%	2%	2vg8_A mol	
6	CsUGTE3	520 bits (1340)	E-147	56%	74%	2%	2vg8_A mol	
7	CsUGTE4	526 bits (1356)	E-149	57%	75%	1%	2vg8_A mol	
8	CsUGTE5	561 bits (1446)	E-159	60%	76%	1%	2vg8_A mol	Hydroquinone
9	CsUGTE6	592 bits (1526)	E-169	62%	77%	0%	2vg8_A mol	glucosyltransferase
10	CsUGTE7	595bits (1533)	E-169	62%	78%	1%	2vg8_A mol	
11	CsUGTE8	589 bits (1518)	E-168	62%	77%	1%	2vg8_A mol	
12	CsUGTE9	517 bits (1332)	E-146	55%	72%	1%	2vg8_A mol	
13	CsUGTE10	369 bits (948)	E-102	52%	68%	3%	2vg8_A mol	
14	CsUGTG2	553bits (1425)	E-157	54%	72%	1%	2pq6_A mol	
15	CsUGTG3	584 bits (1506)	E-166	58%	74%	1%	2pq6_A mol	
16	CsUGTG4	375 bits (964)	E-104	58%	76%	1%	2pq6_A mol	
17	CsUGTG5	569 bits (1467)	E-162	56%	74%	1%	2pq6_Amol	Multifunctional
18	CsUGTG6	588 bits (1257)	E-138	50%	66%	4%	2pq6_Amol	(iso)flavonoid
19	CsUGTG8	549 bits (1415)	E-156	55%	73%	1%	2pq6_A mol	glycosyltransferase
20	CsUGTG9	562 bits (1448)	E-160	55%	72%	1%	2pq6_Amol	
21	CsUGTG12	480 bits (1236)	E-135	55%	74%	1%	2pq6_Amol	
22	CsUGTG13	572 bits (1474)	E-163	56%	74%	1%	2pq6_A mol	

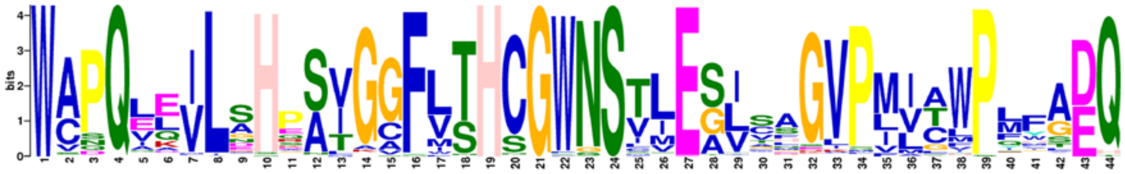

Supplementary Fig. S1. PSPG motif of CsUGTs.

The description of PSPG motif of all CsUGTs. Motif elicitation was performed by MEME online (http://meme.nbcr.net/meme/cgi-bin/meme.cgi)

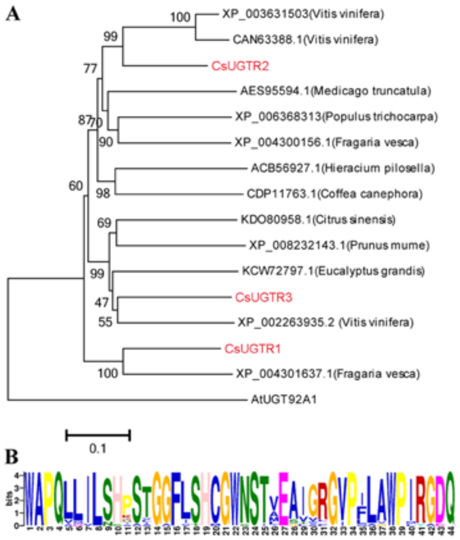

Supplementary Fig. S2. Phylogenetic analysis and PSPG motif of the group R.

(A) Phylogenetic analysis of group R. The phylogenetic tree was constructed as described in Fig. 2 and rooted with *A. thaliana* UGT92A1 from group M. Amino acid sequences used were three UGT members from *C. sinensis* and 17 putative UGTs from other plant species.

(B) The description of PSPG motif of group R. Motif elicitation was performed by MEME online (http://meme.nbcr.net/meme/cgi-bin/meme.cgi).

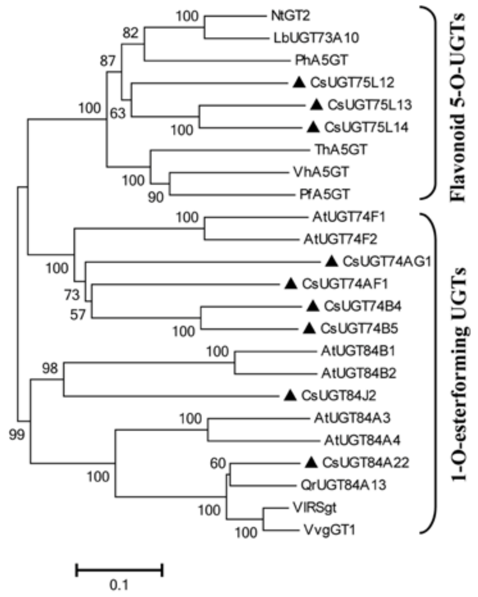

Supplementary Fig. S3. The position divergence of glycosylation for nine CsUGTs in group L and several UGTs from other plant species. Genebank accession numbers for these sequences are NtGT2(BAB88935.1); LbUGT73A10(BAG80544.1); PhA5GT(BAA89009); CsUGT75L12(KP682364); CsUGT75L13(KP682365); CsUGT75L14(KP682366); ThA5GT(BAC54093); VhA5GT(BAA36423); PfA5GT(BAA36421); AtUGT74F1(AEC10333.1); AtUGT74F2(AEC10331.1); CsUGT74AG1(KP682370); CsUGT74AF1(KP682369); CsUGT74B4(KP682367); CsUGT74B5(KP682368); AtUGT84B1(AAB87119); AtUGT84B2(AAB87106); CsUGT84J2(KP682363); AtUGT84A3(NP_193284.1); AtUGT84A4(AEE83611.1); CsUGT84A22(KP682362); QrUGT84A13(KF527849); VLRSgt(ABH03018); VvgGT1(AEW31187.1).

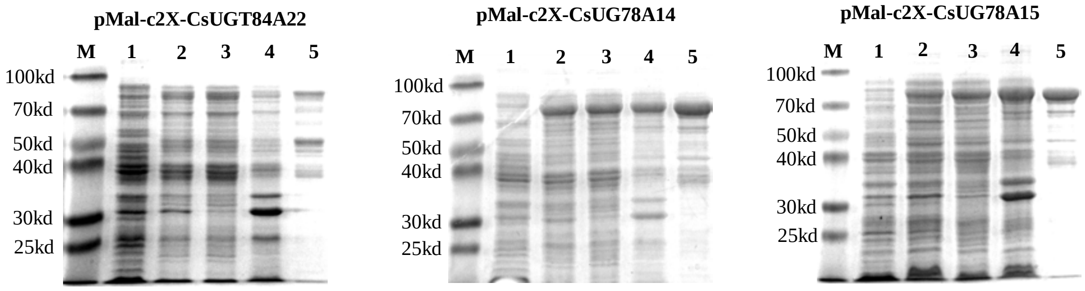

Supplementary Fig. S4. SDS-PAGE analysis of protein extracts from *E. coli* expressing CsUGT-maltose binding protein fusion. M, protein molecular weight marker; lane 1, uninduced cells; lane 2, induced cells; lane 3, supernatant protein of induced cells; lane 4, protein precipitation of induced cells; lane 5, purified protein eluted from amylose column with maltose.

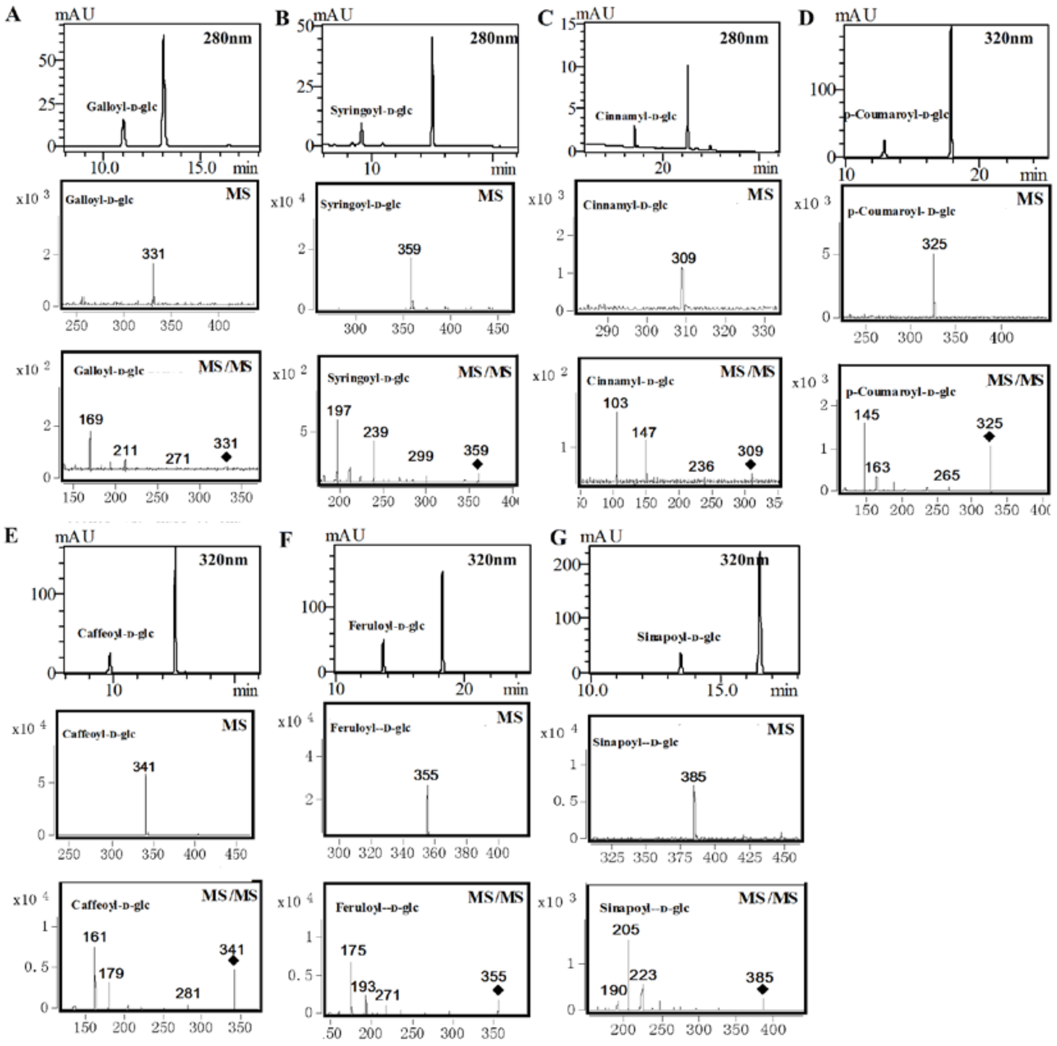

Supplementary Fig. S5. HPLC charts (up) , Mass spectrum (middle) and MS2 spectrum (down) of the enzymatic products catalyzed by rCsUGT84A22 with gallic acid (A), syringic acid (B), cinnamic acid (C), *p*-coumaric acid (D), caffeic acid (E), ferulic acid (F), sinapic acid (G) as substrates.

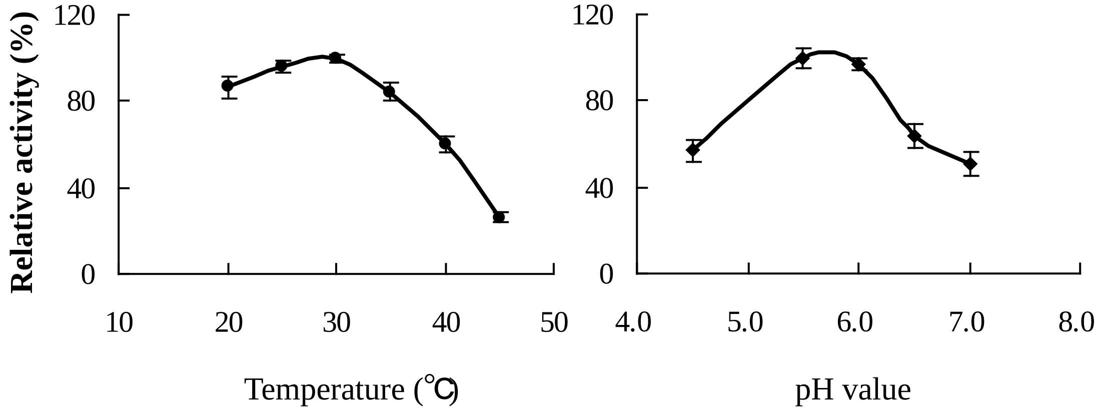

Supplementary Fig. S6. Temperature and pH value optimization for the activity of rCsUGT84A22 with gallic acid and UDP-glucose as substrates. Temperatures were tested from 20 to 45 ℃ at pH 5.5, and pH values were tested with pH 4.5~7.0 at 30 ℃. Data indicated the means and standard deviations from three triplicate assays.

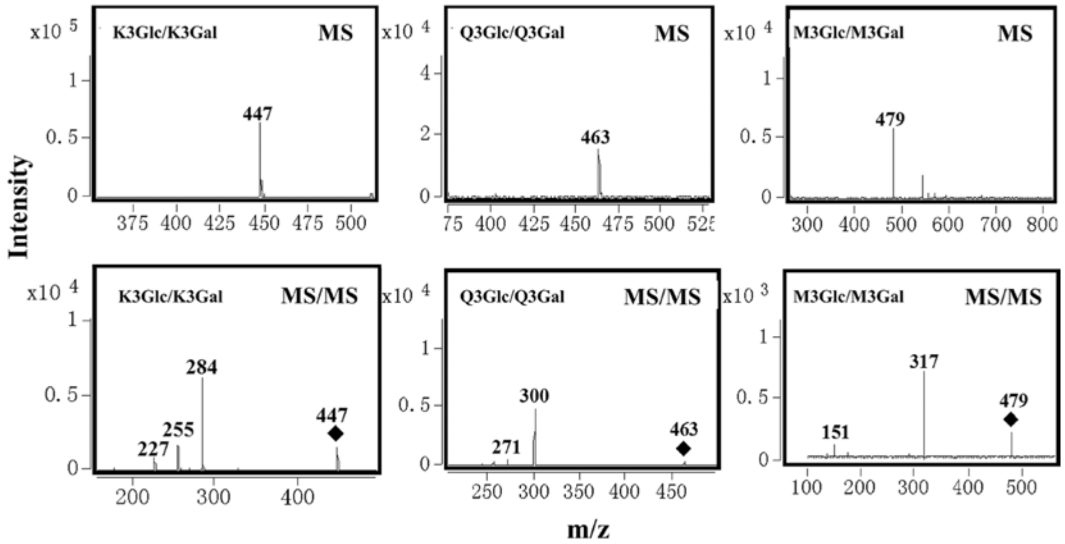

Supplementary Fig. S7. Mass and MS/MS spectrum of enzymatic products of rCsUGT78A14 and rCsUGT78A15 enzymes. Mass (upper panels) and MS/MS (lower panels) spectrum of rCsUGT78A14 and rCsUGT78A15 enzymatic products, including kaemferol-3-*O*-glucosides/ 3-*O*-galactosides (left), quercetin-3-*O*-glucosides/ 3-*O*-galactosides (middle), myricetin-3-*O*-glucosides/ 3-*O*-galactosides (right).
